# Characterization of New TRPM8 Modulators in Pain Perception

**DOI:** 10.3390/ijms20225544

**Published:** 2019-11-07

**Authors:** Carmen De Caro, Claudia Cristiano, Carmen Avagliano, Alessia Bertamino, Carmine Ostacolo, Pietro Campiglia, Isabel Gomez-Monterrey, Giovanna La Rana, Oreste Gualillo, Antonio Calignano, Roberto Russo

**Affiliations:** 1 Department of Pharmacy, University of Naples Federico II, 80131 Naples, Italy; carmen.decaro@unina.it (C.D.C.); cla.cristiano@gmail.com (C.C.); carmen.avagliano@gmail.com (C.A.); carmine.ostacolo@unina.it (C.O.); imgomez@unina.it (I.G.-M.); gilarana@unina.it (G.L.R.); calignan@unina.it (A.C.); 2 Department of Science of Health, School of Medicine and Surgery, University of Catanzaro, 88100 Catanzaro, Italy; 3 Department of Pharmacy, University of Salerno, 84084 Fisciano, Italy; abertamino@unisa.it (A.B.); pcampiglia@unisa.it (P.C.); 4 SERGAS (Servizo Galego de Saude) and IDIS (Instituto de Investigación Sanitaria de Santiago), The NEIRID Group (Neuroendocrine Interactions in Rheumatology and inflammatory Diseases), Santiago University Clinical Hospital, 15706 Santiago de Compostela, Spain; oreste.gualillo@sergas.es

**Keywords:** TRPM8, orofacial pain, neuropathic pain, local application, systemic administration

## Abstract

Background: Transient Receptor Potential Melastatin-8 (TRPM8) is a non-selective cation channel activated by cold temperature and by cooling agents. Several studies have proved that this channel is involved in pain perception. Although some studies indicate that TRPM8 inhibition is necessary to reduce acute and chronic pain, it is also reported that TRPM8 activation produces analgesia. These conflicting results could be explained by extracellular Ca^2+^-dependent desensitization that is induced by an excessive activation. Likely, this effect is due to phosphatidylinositol 4,5-bisphosphate (PIP2) depletion that leads to modification of TRPM8 channel activity, shifting voltage dependence towards more positive potentials. This phenomenon needs further evaluation and confirmation that would allow us to understand better the role of this channel and to develop new therapeutic strategies for controlling pain. Experimental approach: To understand the role of TRPM8 in pain perception, we tested two specific TRPM8-modulating compounds, an antagonist (IGM-18) and an agonist (IGM-5), in either acute or chronic animal pain models using male Sprague-Dawley rats or CD1 mice, after systemic or topical routes of administration. Results: IGM-18 and IGM-5 were fully characterized in vivo. The wet-dog shake test and the body temperature measurements highlighted the antagonist activity of IGM-18 on TRPM8 channels. Moreover, IGM-18 exerted an analgesic effect on formalin-induced orofacial pain and chronic constriction injury-induced neuropathic pain, demonstrating the involvement of TRPM8 channels in these two pain models. Finally, the results were consistent with TRPM8 downregulation by agonist IGM-5, due to its excessive activation. Conclusions: TRPM8 channels are strongly involved in pain modulation, and their selective antagonist is able to reduce both acute and chronic pain.

## 1. Introduction

Transient receptor potential (TRP) channels are a group of ion channels belonging to the voltage-gated superfamily, including voltage-gated K^+^, Na^+^, and Ca^2+^ channels and cyclic nucleotide-gated channels [1]. TRP channels are expressed in many tissues and cells, with different functions in neurobiology and grouped into several subfamilies. Among them, TRPM subfamily is the most enigmatic [2], with TRPM8 one of the best-studied members. TRPM8 is a non-selective cation channel activated by noxious, cold, and chemical compounds such as menthol and icilin [3,4]; it is expressed in a subpopulation of cold-sensitive dorsal root ganglion (DRG) neurons, in sensory nerves, and it has also been found in the bladder and male genital tract [5,6]. Several studies indicate the contribution of this channel in the pathophysiology of cold allodynia and nociception [7]. The role of TRPM8 in modulating pain perception has been largely evaluated by means of TRPM8-deficient (*Trpm8*^−/−^) mice [8,9]. Moreover, an increase of TRPM8 expression in different animal models such as neuropathy induced by oxaliplatin [10] and by streptozotocin-induced diabetes [11] is documented, confirming its role in the somatic sensory nervous system response [12] and underlining its function in pain management [13]. In support of these findings, we have recently shown that novel TRPM8 inhibitors remarkably reduced cold and mechanical allodynia in acute and chronic pain models [14]. These results clearly indicate that TRPM8 inhibition has an analgesic effect; however, other studies showed in some animal models of nerve injury, that peripheral and central activation of TRPM8 is also followed by analgesia [15]. Moreover, functional and anatomical studies also demonstrated a direct role of TRPM8 in inflammation; however, this needs to be further elucidated [16]. Several other studies showed apparently controversial results. For example, selective TRPM8 inhibitors were able to reduce allodynia and hyperalgesia, suggesting that this channel significantly contributes to neuropathic pain modulation [14,17]. On the other hand, some other evidence has established the analgesic properties of TRPM8 agonists [18,19]. Many theories have been proposed to explain these conflicting data; among these, the hypothesis that has found greater consensus is the negative modification of the receptor. It is widely known that the hyperstimulation of a membrane receptor can induce an important alteration of its functionality, leading to inactivation. The TRPM8 receptor-channel can undergo desensitization, probably due to a depletion of the second messenger, PIP2, which influences both the channel activity and the membrane potentials [20,21]. Therefore, the development of antagonists would be preferred for a possible therapeutic exploitation; however, the study of selective TRPM8 agonists is to be considered in order to better understand the complex phenomenon of desensitization [17].

**Chart 1:**
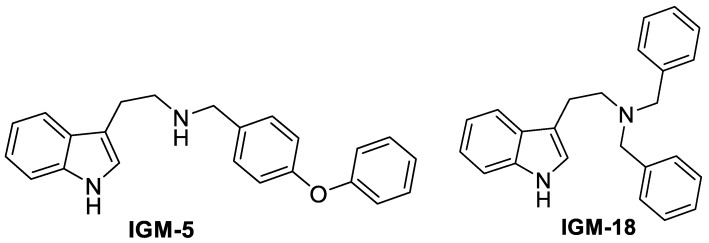
Molecular structures of IGM-5 and IGM-18, TRPM8 agonist and antagonist, respectively.

To better understand the role of this channel in pain perception, in this study, we tested two compounds, an agonist and an antagonist of TRPM8 receptor (Chart 1), previously evaluated in vitro [22], in either acute or chronic pain models using both systemic and local routes of administration.

## 2. Results

### 2.1. Effect of IGM-18 on Formalin-Induced Orofacial Pain

Formalin-induced neurogenic inflammation is a typical pain model used for evaluating the antinociceptive properties of drugs. Injection of formalin produces a significant edema, with long-lasting secondary hypersensibility, from 12 up to 72 h. In the present work, formalin was injected into the upper lip region of rats in order to produce orofacial allodynia, as previously done [14]. Then, the effect of IGM-18 on this formalin induced orofacial pain model was evaluated.

**Figure 1:**
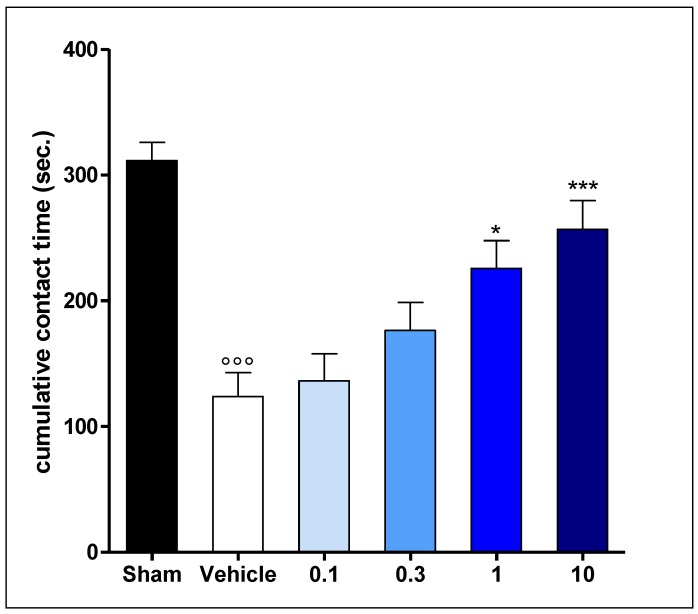
Effect of vehicle and IGM-18 in formalin-induced orofacial pain hypersensibility. After 12 h from formalin injection, rats were treated with vehicle (white column) and IGM-18 (0.1–10 mg/kg; blue columns); test was done 1 h following systemic administration. The sham group represents the measurement before formalin injection. The cumulative contact time was counted for 10 min. Data are shown as mean ± SEM of 6 animals per group. °°° *p* < 0.001 vs. sham group; * *p* < 0.05 and *** *p* < 0.001 vs. vehicle.

We observed that 12 h after formalin injection into the upper lip region of rats, the vehicle-treated group showed a significant reduction of contact time by mechanical stimulus (Figure 1, white bar; °°° *p* < 0.001 vs. the respective basal group) compared to sham rats. Systemic IGM-18 pre-treatment (1 h before) decreased formalin-induced pain in a dose-dependent manner: Indeed, at 1 mg/kg (* *p* < 0.05) and 10 mg/kg (*** *p* < 0.001), it significantly reduced the contact time compared to the vehicle group (Figure 1), while no significant effect was observed using doses of 0.1 and 0.3 mg/kg (Figure 1).

### 2.2. Effect of TRPM8 Antagonist (IGM-18) in Chronic Constriction *Injury* (CCI)-Induced Neuropathic Pain

**Figure 2:**
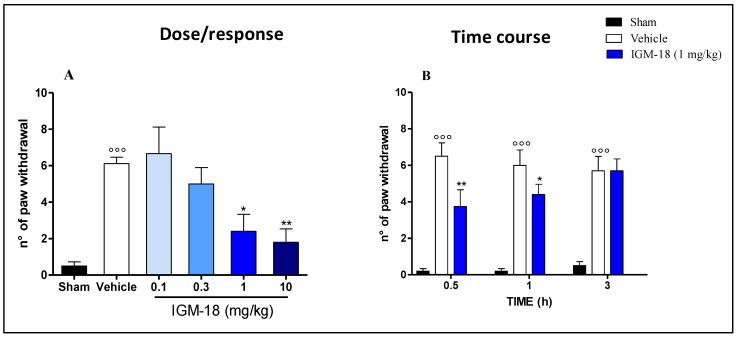
(**A**) Dose-response effect of IGM-18 (0.1–10 mg/kg/i.p) on CCI-induced neuropathic pain. IGM-18 (blue columns) and vehicle (white column) were administered intraperitoneally on day 14 after ligation, 1 h before the test. The sham group did not receive ligation (black columns). Data are shown as mean ± SEM of 6 animals per group. °°° *p* < 0.001 vs. sham group; * *p* < 0.05 and ** *p* < 0.01 vs. vehicle. (**B**) Time course effect of vehicle (white column) and IGM-18 (1 mg/kg; blue columns) following systemic administration. Drug was administered intraperitoneally 0.5, 1, and 3 h before test. Data are shown as mean ± SEM of 6 animals per group. °°° *p* < 0.001 vs. sham group; * *p* < 0.05 and ** *p* < 0.01 vs. vehicle.

**Figure 3:**
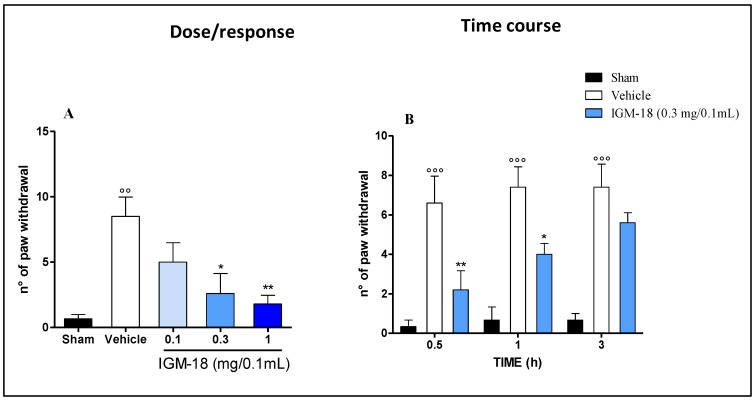
(**A**) Dose-response effect of IGM-18 (0.1-1 mg/0.1 mL/paw) on CCI-induced neuropathic pain. IGM-18 (blue columns) and the vehicle (white column) were administered locally on day 14 after ligation, 0.5 h before the test. The sham group did not receive ligation (black columns). Data are shown as mean ± SEM of 6 animals per group. °° *p* < 0.01 vs. sham group; * *p* < 0.05 and ** *p* < 0.01 vs. vehicle. (**B**) Time course effect of vehicle (white column) and IGM-18 (0.3 mg/0.1 mL/paw; blue columns) in cold allodynia induced by CCI after local administration. Tests were performed at 0.5, 1, and 3 h after IGM-18 or vehicle application. Data are shown as mean ± SEM of 6 animals per group. °°° *p* < 0.001 vs. sham group; * *p* < 0.05 and ** *p* < 0.01 vs. vehicle.

The CCI model is a commonly used model of neuropathic pain. In this experiment, on day 14, we evaluated the dose-dependent effect of IGM-18 following a single systemic administration, 1 h before the measurement of cold sensitivity. The vehicle-treated group showed a significant increase of cold responses compared to the sham group (Figure 2A, white bars; °°° *p* < 0.001). IGM-18 induced a reduction of allodynia; this effect was statistically significant at the highest doses, 1 and 10 mg/kg (Figure 2A; ** *p* < 0.01 and * *p* < 0.05 vs. CCI-vehicle-treated mice), while no activity was observed at the lower doses of 0.1 and 0.3 mg/kg. Subsequently, we evaluated time course using the first active dose, 1 mg/kg. As reported in Figure 2B, animals treated with the vehicle showed a significant cold allodynia compared to sham mice (Figure 2B, white bars; °°° *p* < 0.001), while systemic IGM-18 (1 mg/kg) administration produced a marked reduction of the number of paw withdrawals at 0.5 h and 1 h post-dose (Figure 2B; ** *p* < 0.01 and * *p* < 0.05 vs. CCI-vehicle-treated mice); no effect was observed at 3 h post-dose and on the contralateral (unlighted) paw (data not shown). In another set of CCI experiments, the activity of TRPM8 antagonist following local administration on the paw was tested. Three different doses were used (0.1–1 mg/100 μL/paw). As reported in Figure 3A, animals treated with the vehicle showed a significant cold allodynia compared to sham rats (Figure 3A, white bar; °° *p* < 0.01 vs. sham mice). IGM-18 reduced allodynia in a dose-dependent manner. Indeed, the dose of 0.3 mg/paw produced a significant antiallodynic effect that became more marked using the higher doses of IGM-18 (1 mg/100 μL/paw). No effect was observed using the lowest dose (0.1 mg/100 μL/paw) (Figure 3A; ** *p* < 0.01 and * *p* < 0.05 vs. CCI-vehicle-treated mice). Figure 3B reports the time course (0.5, 1, and 3 h after local application) using the first active dose. Local IGM-18 (0.3 mg/100 μL/paw) application produced a significant reduction of the number of paw withdrawals at 0.5 and 1 h post-application (Figure 3B; ** *p* < 0.01 and * *p* < 0.05 vs. CCI-vehicle-treated mice); no effect was observed at 3 h post-application.

### 2.3. Effect of TRPM8 Agonist (IGM-5) on CCI-Induced Neuropathic Pain

**Figure 4:**
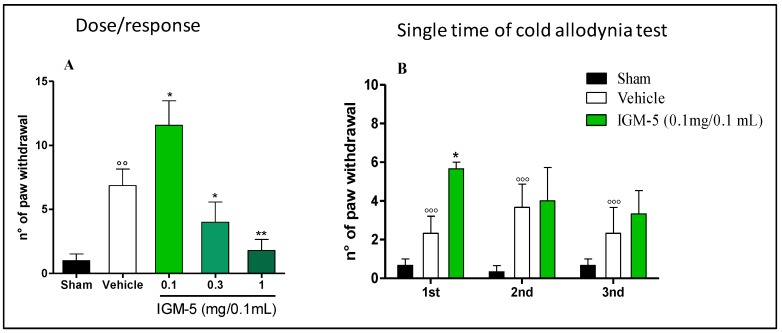
(**A**) Dose-response effect of IGM-5 (0.1–1 mg/0.1 mL/paw) on CCI-induced neuropathic pain. The vehicle (white column) and IGM-5 (green columns) were administered locally on day 14 after ligation 0.5 h before the test. The sham group did not receive the ligation (black columns). Data are shown as mean ± SEM of 6 animals per group. °° *p* < 0.01 vs. sham group; * *p* < 0.05 and ** *p* < 0.01vs. vehicle. (**B**) Time course effect of the vehicle (white column) and IGM-5 (0.1 mg/0.1 mL/paw; green columns) on cold allodynia induced by CCI after local administration. Allodynia responses were evaluated after the first (1st), the second (2nd), and the third (3nd) acetone application. Data are shown as mean ± SEM of 6 animals per group. °°° *p* < 0.001 vs. sham group; * *p* < 0.05 vs. vehicle.

Based on the aforementioned results, we investigated the effects of TRPM8 agonist (IGM-5) local application on the CCI mouse model of cold allodynia. Three different doses were used (0.1–0.3 and 1 mg/100 μL). Animals treated with the vehicle showed a significant cold allodynia compared to sham mice (Figure 4A, white bar; °° *p* < 0.01 vs. sham mice). IGM-5 induced hypersensitivity using the lowest dose (0.1 mg) compared to vehicle-treated mice, while the intermediate and highest doses (0.3 and 1 mg, respectively) produced a significant reduction of cold responses (** *p* < 0.01 and * *p* < 0.05 vs. vehicle-treated mice). Using only the lowest dose, we measured paw withdrawal numbers after each one of the three consecutive acetone applications (reported in figure as “single phase”). As shown in Figure 4B, acetone failed to produce a significant effect of cold allodynia after the second and the third applications, while a significant increase of numbers of paw withdrawal were detected after the first acetone exposure (* *p* < 0.05 vs. vehicle-treated mice). IGM-5 (0.1–10 mg/kg) was also tested by systemic administration, but no significant effect was observed at all used doses (data not shown).

### 2.4. Effect of IGM-18 on Icilin-Induced Wet-Dog Shake (WDS)

**Figure 5:**
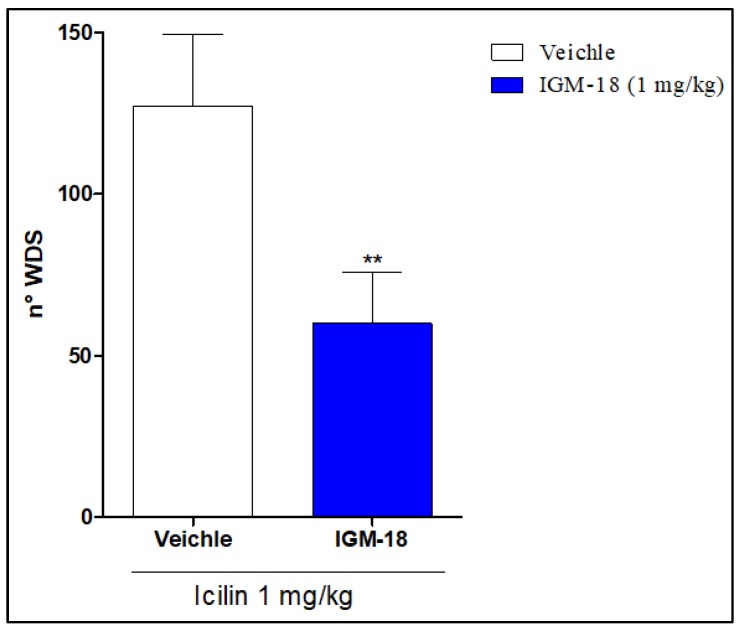
Effect of IGM-18 on icilin-induced wet-dog shake (WDS). Rats were pre-treated with the vehicle (white column) and IGM-18 (1 mg/kg; blue column), after 0.5 h icilin was injected (1 mg/kg, i.p.) and the number of WDS were counted for 30 min. Data are shown as mean ± SEM of 6 animals per group. ** *p* < 0.01 vs vehicle group.

Subsequently, we evaluated the ability of TRPM8 antagonist to block the spontaneous wet-dog shake (WDS) induced by icilin. For this propose, IGM-18 was administrated 1 h before icilin (1 mg/kg, i.p.) and WDS were recorded for 30 min. In the vehicle-treated group, an average of 127 shakes was counted (Figure 5, white column). The pre-treatment with IGM-18 (1 mg/kg) significantly decreased by 47% the number of icilin-induced WDS (Figure 4; ** *p* < 0.01 vs. vehicle-treated rats).

### 2.5. Effect of TRPM8 Modulators on Body Temperature

**Figure 6:**
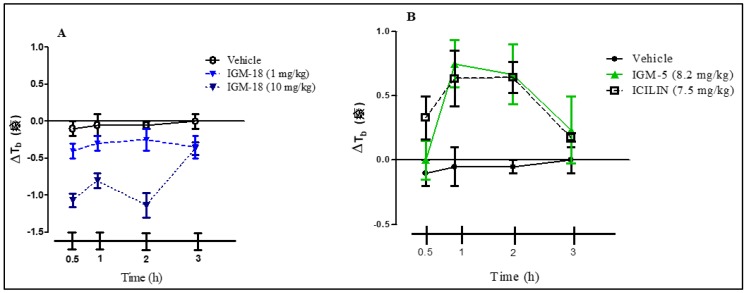
(**A**) Effect of IGM-18 (1 and 10 mg/kg) on body temperature (Tb). Tb was measured at time 0 (before treatment) and 0.5, 1, 2, and 3 h post-drug administration. (**B**) Effect of Icilin (7.5 mg/kg) and equimolecular dose of IGM-5 Icilin (8.2 mg/kg) on body temperature (Tb). Tb was measured at time 0 (before treatment) and 1, 2, and 3 h post-drug administration. Data are shown as mean [differences between temperature after (Tpost dose) and before treatment (Tbasal)] ± SEM of 6 animals per group.

Finally, we evaluated the effects of IGM-18 and the IGM-5 on body temperature (Tb). The acute treatment with IGM-18 at the highest dose (10 mg/kg) produced a significant decrease of Tb at different time points; specifically, of −1 °C at 0.5 h, −0.8 °C at 1 h, −1.2 °C at 2 h, without a significant reduction at 3 h (Figure 6A, * *p* < 0.05 vs. vehicle). As expected, intramuscular administration of icilin (7.5 mg/kg), used as positive control, increased Tb values with a maximum of 0.6 °C, 2 and 3 h after treatment (Figure 6B, black square; * *p* < 0.05 vs. vehicle group). Similarly, intramuscular administration of TRPM8 agonist (IGM-5), used at equimolecular dose of icilin (8.2 mg/kg), produced a significant increase of Tb; specifically, of +0.1 °C at 0.5 h, +0.8 °C at 1 h, and +0.6 °C at 2 h, while no effect was detectable at 3 h (Figure 6B, * *p* < 0.05 vs. vehicle group).

### 2.6. Selectivity Studies

Several other TRP channels have been described as involved in pain perception, particularly TRPV1 and TRPA1 that share high homology with TRPM8. This is because the prototypical TRPM8 agonist menthol [23,24], and some canonical TRPM8 antagonists such as BCTC [25], are also able to modulate TRPV1 and TRPA1 channels. Thus, selectivity represents one of the main issues in the development of TRPM8 modulators [26]. On the other side, it has also been reported that NaV1.7 channels are involved in acute mechanical, thermal, and chemical noxious stimuli perception. For these reasons, IGM-18 and IGM-5 were examined for their selectivity by further investigation using fluorescence imaging. As shown in Table 1, the two compounds proved to be highly selective for TRPM8, since IGM-5 was mostly inactive on the three selected targets (with a really weak antagonism on NaV1.7). Similarly, IGM-18 was inactive as antagonist on the selected targets, showing only a weak agonistic activity on TRPA1 with a ten-fold selectivity for TRPM8.

## 3. Discussion

The discovery of the transient receptor potential cation channels in primary sensory neurons has changed our understanding about pain perception; in particular, regarding cutaneous temperature detection. It is well known that TRPM8 is expressed by a small population of cold-sensitive sensory neurons and is activated at low temperatures and by the cooling compound menthol or icilin [3,5]. TRPM8 has also been involved in the painful hypersensitivity that is a worrying symptom in inflammation and neuropathy. A large body of studies has demonstrated that TRPM8 antagonists are effective in reducing thermal and mechanical hyperalgesia, suggesting for this channel an important role in pain perception [8,14,17]. Therefore, it would be greatly helpful to patients with acute and chronic pain to have a compound able to control these symptoms. Here, we tested two new TRPM8 modulators, IGM-5 and IGM-18, a selective agonist and antagonist, respectively, to characterize their contribution on pain, using both systemic and local routes of administration. Conflicting studies have shown that TRPM8 is involved in pain. Data on knockout animals have shown that TRPM8 is involved in cold sensation induced by drop of acetone or chemical compounds, such as menthol and icilin [9,27,28]. In this study, we demonstrated that both direct antagonism and the excess dose of agonist, responsible of receptor desensibilization [29], led to the inactivation of this channel that is necessary to decrease pain perception. Indeed, IGM-18, a selective TRPM8 antagonist, following systemic administration or local application, is able to reduce pain. In line with our data, it is reported that TRPM8 is involved in orofacial and visceral (colonic) pain [8,18,30,31,32], and that its activation by menthol causes irritation in some patients [18]. In our model of orofacial pain induced by formalin, IGM-18 was able to reduce the contact time to mechanical stimulus, confirming the pivotal role of TRPM8 in acute pain [14]. Moreover, TRPM8 antagonist was also used in a chronic pain model, such as neuropathy. It is well known that there is an upregulation of this channel in DRG, after chronic constriction injury of the sciatic nerve (CCI) or chemotherapy-induced neuropathic pain [33,34]. In our protocol, IGM-18 at the dose of 1 mg/kg reduced cold hyperalgesia induced by CCI, and these effects were significant after both local application and systemic administration. A previous in vitro characterization [22], together with the selectivity assays performed in the present study, highlight the remarkable selectivity of IGM-18 on channel TRPM8. Interestingly, it has been reported that TRPM8 activator reduced mechanical and thermal nociception in the CCI model through a central modulation [15], and these analgesic effects are supported by a study using TRPM8 knock-out mice [28]. Moreover, menthol is widely used as a topical analgesic and inhaled antitussive with respiratory counterirritant properties. Menthol and TRPM8-selective agonists have been shown to suppress nocifensive responses to several acute pain stimuli, including capsaicin, acrolein, and acid [18]. Our data showed that systemic IGM-5 administration did not modify paw withdrawal latency in mice that underwent CCI (data not shown). By contrast, local application showed opposite effects, i.e., lower dose-induced pain, while high doses showed a significant antiallodynic activity. This conflicting data could be explained through the alteration of TRPM8 activity, since it has been demonstrated that the activation of this channel is followed by extracellular Ca^2+^-dependent desensitization, probably due to the PIP2 depletion that leads to the reduction of TRPM8 channel activity, accompanied by a shifting voltage dependence towards more positive potentials [20,21]. Overall, it seems likely that TRPM8 is normally expressed in a distinct population of cool-responsive afferents, and also in some nociceptors [3]. Moreover, other behavioral studies using icilin or menthol have also pointed out that agonist activation of TRPM8 may cause sensitization or desensitization/inhibition of responses to mechanical stimuli under naïve conditions [35,36,37] or after injury [15,18,35]. Furthermore, it has been demonstrated that TRPM8-mediated analgesia was selectively reversed by intrathecal administration of Group II/III mGluR antagonists and mimicked by agonists, and ionophoresis of a Group III mGluR antagonist reversed the inhibitory effect of icilin on sensitized single neurons [15]. In fact, it is possible that the TRPM8 activation produces a modulation of glutamate release, inducing an alteration in pain perception [38,39].

However, given that the aim of a good symptom-controlling drug would be to reduce the hypersensitivity without abolishing normal thermosensation, this may not be a completely undesirable effect. It seems that TRPM8-expressing afferents affect thermoregulatory responses to both chemical and thermal stimuli, although the exact neurological mechanism remains unknown [40,41]. Because of this evidence, and reports underling that TRPV1 antagonists have undesired thermoregulatory effects [42], we thought it possible that a TRPM8 modulator would also affect thermoregulation. Indeed, it is suggested that TRPM8 plays a key role in thermoregulation, with the stimulation of skin afferents both with chemical and cooling agonists [40]. Here, we have confirmed that IGM-5 induced an increase in body temperature, as well as icilin, a chemical TRPM8 agonist [43], an effect that is TRPM8-dependent due to the lack of modulation on TRPA1 channels, as shown by in vitro selectivity studies [3,30]. By contrast, systemic IGM-18 administration reduced body temperature in a dose-dependent manner, and it is noteworthy that the effective dose of 1 mg/kg did not produce a significant alteration of body temperature. This suggests that the reduction of pain is due to modulation of pain pathways, without any alteration of body temperature. Further experiments are needed to additionally characterize these two novel TRPM8 channel modulators.

## 4. Materials and Methods

### 4.1. Animals

Behavioral experiments were performed on male Sprague-Dawley rats (300–310 g Charles Rivers-Italy) or male CD1 mice (25–30 g Charles Rivers-Italy). They were housed in the animal care facility of the Department of Pharmacy, University of Naples Federico II. Animals were acclimated to their environment for 1 week and had ad libitum access to standard rodent chow pellets VRF1 (purchased from Special Diet Service-SDS, UK, LBS Biotech) and water. All behavioral tests were performed between 9:00 AM and 1:00 PM, and the animals were only tested once. Procedures involving animals and their care were carried out in conformity with international and national law and policies (EU Directive 2010/63/EU for animal experiments). The procedures reported here were approved by the Institutional Committee on the Ethics of Animal Experiments (CSV) (protocol no. 0084607, date 3 October 2014.) of the University of Naples “Federico II” and by the “Ministero Della Salute”, At the end of the experiments, animals were euthanized by CO_2_ overdose. As suggested by the animal welfare protocol, all efforts were made to minimize animal suffering and to use only the number of animals necessary to produce reliable scientific data.

### 4.2. Drug Treatments

During this study, IGM-18 was administered systemically and locally, while IGM-5 only by local administration (excluding the temperature test, using the local equimolecular by systemic administrations). IGM-5 and IGM-18 in local treatments were solubilized in absolute ethanol and used at dose of 0.1, 0.3, and 1 mg/100 μL of ethanol and applied on operated paw (*n* = 6 for each experimental group). The vehicle group was treated with absolute ethanol only. During systemic administration, two drugs were dissolved in PEG400 10% (v/v), TWEEN 80 5% (v/v), and sterile saline and used at different doses (0.1, 0.3, 1, and 10 mg/kg). The vehicle group was treated with PEG, TWEEN, and sterile saline only.

### 4.3. Induction of Orofacial Pain

Orofacial pain hypersensitivity was performed on rats and was induced by formalin, according to the method described by [44] with some modifications [14]. Briefly, 2.5% formalin (0.1 mL) was injected into the upper lip, just lateral to the nose using a 27G sterile needle mounted on a 1 mL syringe; the needle was inserted into the lip close to the border between hairy and glabrous skin and advanced up to the hilt towards the vibrissa pad. Thus, the formalin solution spread-out within the upper lip and the rostral half of the vibrissa pad, inducing a stereotypical response characterized by two distinct phases: An initial neurogenic phase (0–3 min) and a second inflammatory phase (10–30 min). Differently from [44], we evaluated pain hypersensitivity by mechanical and cold stimuli developed 24 h after formalin injection using the Orofacial Stimulation Test (Ugo Basile, Italy).

### 4.4. Orofacial Stimulation Test

The orofacial stimulation test system measures hypersensitivity of the trigeminal area. Initially, animals underwent 5 days of training, utilizing the orofacial stimulation test after a 12 h-period of food deprivation (fasting period). Rats were placed in a standard rat cage with a plastic divider to create two rooms. In the anterior room of the cage, there was an Ugo Basile apparatus with a drinking window for the rat’s head to enter and acquire a reward (milk and chocolate) located on the opposing aspect of the drinking window. During the training period, each rat was individually placed in the posterior room for 10 min to familiarize with its environment; subsequently, the drinking window was opened, and the rat was monitored for 10 min to allow it to drink the milk and chocolate [45]. On the day of the behavioral assessment, mechanical or thermal stimulation was induced by the insertion of an appropriate module in the drinking window (not present during the training period). During this time, rats received approximately 3–5 stimulations prior to formalin (basal) and 15–20 stimulations 24 h after formalin injection, for both mechanical and thermal stimulator. Similar to the adaptation training, the mechanical module experiment was preceded by a 12-h fasting period, 10 min for the rats to familiarize with the testing environment, and a subsequent 10 min for behavioral assessments.

### 4.5. Mechanical Stimulator

Experiments were performed utilizing the mechanical model hat, which consisted of a cassette with 10 tungsten wires placed 3 mm apart from each other and 8 mm from the opening hole to the cassette held to produce a bending force from the drinking window of the apparatus. The animal’s face contacted the tungsten wires of the mechanical module as the rat projected its head through the hole in the apparatus in order to drink milk and chocolate located on the exterior aspect of the drinking window. In each experiment, a blind observer activated a stopwatch every time the test rat drank milk and chocolate (also defined as “contact time”); when the test rat moved away from the drinking window, the stopwatch was stopped. Data were generated using the total contact time(s) to mechanical stimulus reached in a total testing time of 600 s. Basal data were obtained from the total contact time (reached in a total testing time of 600 s) 24 h before formalin injection.

### 4.6. Chronic Constriction Injury (CCI) Model of Neuropathic Pain

Neuropathic pain behavior was performed on mice and it was induced by ligation of the sciatic nerve, as described previously [46]. Briefly, mice (*n* = 6 each group) were first anesthetized with xylazine (10 mg/kg, i.p.) and ketamine (100 mg/kg, i.p.), and the left thigh was shaved and scrubbed with betadine. Then, a small incision in the middle left thigh (2 cm in length) was performed to expose the sciatic nerve. The nerve was loosely ligated at two distinct sites (spaced at a 2-mm interval) around the entire diameter of the nerve using silk sutures (7–0). The surgical area was closed and finally scrubbed with betadine. In sham-operated animals, the nerve was exposed but not ligated.

### 4.7. Cold Allodynia

Cold sensitivity was measured as the number of foot withdrawal responses after application of cold acetone on dorsal surface of paw after 14 days from CCI. Individual animals were placed in Plexiglas boxes (30 × 30 × 25 cm), a drop of acetone (25 µL) was applied to the dorsal surface of the ligated paw with a syringe connected to a thin polyethylene tube while the animals were standing on a metal mesh. A brisk foot withdrawal response, after the spread of acetone over the dorsal surface of the paw, was considered as a sign of cold allodynia. Three measures of 3 min were done for each paw and expressed in numbers of paw withdrawals. IGM-5 and IGM-18 were tested after both local application and systemic administration. In systemic administration, doses-response (0.1–10mg/kg) and time course (0.5–3 h) were performed. About local application, the results measured were calculated as full time when the values obtained in all the experimental procedure were measured. For IGM-5, the values obtained during the first, second, and third phases (1st, 2nd, and 3rd) were separately expressed, as reported [29]. Moreover, only for IGM-18, a time course (0.5, 1, and 3 h) after topic application was evaluated.

### 4.8. Body Temperature (Tb) Measurement

Rats were placed into an environmental room maintained at a constant temperature of 21 ± 0.3 °C and relative humidity of 55 ± 2%. The animals were allowed to acclimate for 24 h before taking the first temperature reading. Body temperature (Tb) was recorded using a rectal probe for rats (Model RAT2, Ugo Basile, Italy), which was inserted approximately 5 cm from the anus. A digital thermometer (Model BAT-10, Ugo Basile, Italy) was used. Tb was measured before treatment (baseline) and at 0.5, 1, 2, and 3 h following IGM-18 (10 mg/kg, i.m.), IGM-5 (equimolecular dose of the local dose 8.2 mg/kg, i.m), or icilin (7.5 mg/kg, i.m) administration. 

### 4.9. Icilin-Induced “Wet-Dog” Shaking in Rats

Icilin, a TRPM8 agonist, was used to induce shaking in rats [47]. Rats were first habituated to the testing room for 30 min; afterwards, rats were randomized into treatment groups and treated with the vehicle or TRPM8 antagonist (IGM-18 1 mg/kg, i.p.). Icilin was administered i.p. at 1 mg/kg and dissolved in 1% TWEEN 80. The number of intermittent but rhythmic “wet dog-like” shakes (WDS) of neck, head, and trunk in each animal was counted for a period of 30 min following icilin administration.

### 4.10. Fluorescence Imaging Assays

The compound profiling study was carried out by AXXAM (Bresso, Italy) using human TRPA1, TRPV1, and Nav1.7 to determine the agonistic or inhibitory effect of the tested compounds on any of the targets. Experiments were performed using the following cell lines: HEK-293 (ATCC,^,^ CRL-1573), stably transfected with either hTRPA1 or hNav1.7, and CHO-K1, (ATCC, CCL-61) stably transfected with hTRPV1.

HEK-293 cells were grown in EMEM (MEM Eagle Earl’s salts Balanced Salt Solution), supplemented with 5 mL of 200 mM Ultra glutamine, 1, 5 mL of 100X Penicillin/Streptomycin, 50 mL of Fetal Bovine Serum, and 2 mL of 100 mg/mL G418. CHO-K1 cells were grown in Dulbecco’s modified Eagle’s medium-Ham F-12 (DMEM F-12) (1:1) mixture 500 mL supplemented with 5 mL of 100 mM Sodium Pyruvate, 25 mL of 7.5% Sodium Bicarbonate, 6.5 mL of 1 M Hepes, 5 mL of 100X Penicillin/Streptomycin, 50 mL of Fetal Bovine Serum, 0.25 mL of 10 mg/mL Puromycin, 0.5 mL of 100 mg/mL Zeocin. The HEK-293 cells were seeded in 384-well clear bottom black polystyrene walled poly-D-Lys-coated plates (TwinHelix, Rho, Italy), while the CHO-K1 cells in a 384-well clear bottom black walled polystyrene plates, (Thermo Scientific, Waltham, USA). The extracellular solution used was a Standard Tyrode’s buffer composed by 130 mM NaCl, 5 mM KCl, 2 mM CaCl2, 5 mM NaHCO3, 1 mM MgCl2, 20 mM HEPES, pH 7.4, and sterilized by filtration. Compound dilution was performed in 96-well U bottom plates (Thermo Scientific), then compounds were transferred into 384-well V bottom polypropylene barcoded plates (Thermo Scientific). The experimental activities were performed using the EMCCD camera FLIPRTETRA (MDC). For TRPA1 and TRPV1 selectivity assays, cells were seeded at 10,000 cells/well in 384 MTP in complete medium (25 μL/well). Then, 24 h after seeding, culture medium was removed and cells were loaded with 20 μL/well of 0.5X calcium sensitive dye (Fluo-8 NW) in assay buffer. Plates were incubated for 1 h at room temperature in the dark and then injected at 10 μL/well of test compounds and controls at 3X concentration with the FLIPRTETRA, recording the signal of the emitted fluorescence. A second injection of 15 μL/well of 3X reference activator (at ~EC80) with the FLIPRTETRA was performed, recording the signal of the emitted fluorescence. For Nav1.7 selectivity assays, cells were seeded at 15,000 cells/well following the same protocol described above and using a fluorescent membrane potential sensitive dye (Blue) as readout. The reference agonists used were allylisothiocianate for TRPA1 (AITC, 100–0.0316 μM concentration interval, EC80 = 3 μM), Capsaicin for TRPV1 (300–0.1 nM concentration interval, EC80 = 100 nM), and Veratridine for Nav1.7 (300–0.1 μM concentration interval, EC80 = 10 μM). The reference antagonists used were HC-030031 for TRPA1 (100–0.0316 μM concentration interval, EC80 = 100 μM), Capsazepine for TRPV1 (3.16–0.001 μM, concentration interval, EC80 = 3.16 μM), and Tetrodotoxin (TTX) for Nav1.7 (10–0.00316 μM concentration interval, EC80 = 10 μM). 

Compounds were tested against the targets at eight concentrations, intraplate with quadruplicate data points. In order to obtain the eight concentration points of the dose-response curve, a serial dilution protocol in 100% DMSO was performed for each compound by using a 96 U bottom MTP as support. To obtain the 3-fold concentration to be injected on the cell plate, compounds were further diluted in assay buffer, and the final DMSO amount was 0.1%. In order to generate quadruplicate data points, each well of the 96 MTP was then replicated into four wells of a 384 MTP for each compound and for each concentration to be tested. The dilution plates were produced on the same day of the experiment. For data quality and data analysis, the Screener 14.0 software (Genedata, Basel, CH) was used.

### 4.11. Statistical Analysis

In vivo data are presented as mean ± SEM. In the orofacial test, data are presented as cumulative contact time (sec). In CCI experiments, DPA data are presented as paw withdrawal threshold (g), cold allodynia data are presented as number of paw withdrawals. The significance of differences between groups was determined by a one-way repeated measurements ANOVA, followed by a Bonferroni’s post-hoc test. For the body temperatures, the data are expressed as differences between temperature obtained after injection (Tpost dose) and temperature before treatment (Tbasal); results are presented as ΔTb ± SEM. The significance of differences between groups was determined by a one- or two-way repeated measurements ANOVA, followed by a Bonferroni’s post-hoc test.

## Figures and Tables

**Table 1:** Profiling of the compounds on human Nav1.7, TRPA1 and TRPV1ion channels.

Compound	NaV1.7		TRPA1		TRPV1	
***Agonist mode***	Signal at Max ^a^	EC_50_	Signal at Max ^a^	EC_50_	Signal at Max ^a^	EC_50_
IGM-5	0.11	inactive	14.603	inactive	0.4130	inactive
IGM-18	0.133	inactive	58.176	3.9 M	−1.923	inactive
Venatridine	100	2.70 M	NC	NC	NC	NC
AITC	NC	NC	100	1.15 M	NC	NC
Capsaicin	NC	NC	NC	NC	100	22 nM
***Antagonist mode***						
IGM-5	−41.25	>10 M	−29.88	inactive	−29.88	inactive
IGM-18	−19.23	inactive	−51.69	inactive	−7.47	inactive
Tetrodotoxin	−100	9.2 nM	NC	NC	NC	NC
HC-030031	NC	NC	−100	20.7 M	NC	NC
Capsazepine	NC	NC	NC	NC	−100	98 nM

^a^ The signal recorded at the maximum concentration used, assuming that the maximum signal is 100% at the EC100 of the reference agonist and −100% at the EC100 of the reference antagonist.
